# Optimized rapeseed oils rich in endogenous micronutrients ameliorate risk factors of atherosclerosis in high fat diet fed rats

**DOI:** 10.1186/1476-511X-13-166

**Published:** 2014-10-30

**Authors:** Jiqu Xu, Congcong Ma, Ling Han, Hui Gao, Qi Zhou, Mei Yang, Chang Chen, Qianchun Deng, Qingde Huang, Fenghong Huang

**Affiliations:** Department of Product Processing and Nutriology, Oil Crops Research Institute, Chinese Academy of Agricultural Sciences, 2 Xudong Second Road, Wuhan, 430062 P.R. China; Hubei Key Laboratory of Lipid Chemistry and Nutrition, 2 Xudong Second Road, Wuhan, 430062 P.R. China; Department of Nutrition and Food Hygiene, School of Public Health, Tongji Medical College, Huazhong University of Science and Technology, 13 Hangkong Road, Wuhan, 430030 P.R. China; Department of Gastroenterology, The First People’s Hospital of Yichang, The People’s Hospital of China Three Gorges University, 2 Jiefang Road, Yichang, 443000 P.R. China; Department of Gastroenterology, The People’s Hospital of China Three Gorges University, 2 Jiefang Road, Yichang, 443000 P.R. China

**Keywords:** Optimized rapeseed oils, Micronutrients, Atherosclerosis, Oxidant stress, Plasma lipids, Inflammation

## Abstract

**Background:**

Micronutrients in rapeseed such as polyphenols, tocopherols, phytosterols and phospholipids in rapeseed exert potential benefit to atherosclerosis. Some part of these healthy components substantially lost during the conventional refining processing. Thus some new processing technologies have been developed to produce various endogenous micronutrient-enriched optimized rapeseed oils. The aim of this study is to assess whether optimized rapeseed oils have positive effects on the atherosclerosis risk factors in rats fed a high-fat diet.

**Methods:**

Rats received experiment diets containing 20% fat and refined rapeseed oil or optimized rapeseed oils obtained with various processing technologies as lipid source. After 10 weeks of treatment, plasma was assayed for oxidative stress, lipid profiles and imflammation.

**Results:**

Micronutrients enhancement in optimized rapeseed oils significantly reduced plasma oxidative stress, as evaluated by the significant elevation in the activities of CAT and GPx as well as the level of GSH, and the significant decline in lipid peroxidation. Optimized rapeseed oil with the highest micronutrient contents obtained by microwave pretreatment-cold pressing reduced the levels of TG, TC and LDL-C as well as IL-6 and CRP in plasma.

**Conclusions:**

These results suggest that optimized rapeseed oils may contribute to prevent atherogenesis and make them very promising functional food in cardiovascular health promotion.

## Introduction

Cardiovascular disease (CVD) is the leading cause of premature death in most developed and developing countries and it is also an increasingly important source of disability and contributes in large part to the escalating costs of health care. Atherosclerosis, a manifestation of the pathophysiology underlying CVD, constitutes the single most important contributor to this growing burden of this disease. There are definitive evidences to show that oxidant stress [1], lipid abnormalities [2] as well as chronic inflammation [3] have a crucial involvement in both the initiation and the progression of atherosclerosis.

Rapeseed is a major oilseed crop in China and many other countries. It contains high-quality oil which is one of the most common and cheapest vegetable oils for human diet. Rapeseed oil has the exceptionally low amount of saturated fatty acids in all commodity edible oils and high level of monounsaturated fatty acids [4]. Besides, this kind of plant oil is also naturally rich in *α*-linolenic acid and linoleic acid whose ratio are the closest to the optimum to meet the basic requirements of essential fatty acids in the body [5]. In addition to triacylglycerols, rapeseed also contains many healthy bioactive compounds such as phenolic compounds, tocopherols and phytosterols and these endogenous micronutrients have been reported to possess many health benefits or desirable physiological effects in cardiovascular system. For example, by their abilities to scavenge reactive oxygen species (ROS) directly or form complexes with prooxidant metals, these micronutrients possess a potent antioxidant activity and the various bioavailable antioxidants present in rapeseed oil work in concert to upgrade the complex antioxidant network which increase antioxidant capacity higher than that provided by each separate compound [6–8]. Phytosterols have been reported to inhibit cholesterol absorption and thus reduce circulating levels of total (TC) and low density lipoprotein cholesterol (LDL-C) [9]. Previous studies have also shown an independent effect of phenolics improving plasma lipid profiles [10, 11]. Also, all these compounds are known to have antiinflammatory effects [11–13]. The beneficial effects of these inherent micronutrients might contribute to prevent the initiation and development of atherosclerosis. However, the conventional industrial processes (extraction and refining) lead to substantial losses of these cardiovascular protective micronutrients. In order to improve on the desirable components retention and then to develop new healthy oils, some new processing technologies have been developed recently. The aim of this study is to determine the effects of the various endogenous micronutrient-enriched optimized rapeseed oils on atherosclerosis risk factors in rats fed a high-fat diet.

## Materials and methods

### Oils preparation

Rapeseed oils were produced with different technical procedures. The refined rapeseed oil (RRO) was prepared with conventional extraction and refining processing technology. Other three rapeseed oil processing technologies were applied to obtain high levels of endogenous micronutrients: cold pressing (CP), dehulling-cold pressing (DCP) and microwave pretreatment-cold pressing (MPCP). The fatty acid compositions and endogenous micronutrients contents in different rapeseed oil were shown in Tables 1 and 2, respectively.

**Table 1:** **Fatty acid compositions in different rapeseed oils**

Fatty acid (wt.%)	RRO	CP	DCP	MPCP
Palmitic acid (C16:0)	2.413	2.5	2.485	2.44
Stearic acid (C18:0)	3.667	3.769	3.846	3.548
Oleic acid (C18:1)	66.19	66.075	66.287	66.561
Linoleic acid (C18:2)	16.822	16.798	17.088	16.447
Linolenic acid (C18:3)	9.146	9.281	8.463	9.431

**Table 2:** **Endogenous micronutrients contents in different rapeseed oils**

mg/kg oil	RRO	CP	DCP	MPCP
Phenols (in eq., sinapic acid)	8	34	43	645
Of which canolol	ND*	107	167	816
Phytosterols	9592	9902	8834	11027
Tocopherol	461	541	594	600
Phospholipids	22	600	590	1330

*ND, not detected.

### Animals and diets

Forty male Wister rats, initially weighing 150–170 g, were obtained from Vital River Laboratory Animal Center (Beijing, China). The rats were housed individually and maintained at a controlled ambient temperature (24 ± 1°C) under diurnal conditions (light–dark: 08:00–20:00) with access to laboratory chow and tap water ad libitum. After the rats were acclimated for 1 week, animals were randomly divided into four groups of 10 rats each, consisting of RRO, CP, DCP and MPCP groups. The high-fat diet contained 20% casein, 35% maize starch, 15% glucose, 5% cellulose, 3.5% mineral mixture (AIN-93 M), 1% vitamin mixture (AIN-93 M), 0.2% choline bitartrate, 0.3% DL-methionine and 20% fat. The fat in the diet was provided by different rapeseed oils mentioned above. All animals were weighed twice a week and food intake was measured weekly. The animals were cared for in accordance with *the Guiding Principles in the Care and Use of Animals*. The experiment was approved by the Oil Crops Research Institute Council on Animal Care Committee, Chinese Academy of Agricultural Sciences.

### Blood processing

After 10 weeks of treatment, rats were fasted for 16 hours and then killed under anaesthesia, blood was collected in the presence of sodium heparin from the heart immediately. Blood samples were centrifuged at 1500 g (10 min, 4°C) and the plasma was stored at -80°C until analysis.

### Plasma lipids analysis

The plasma triglyeride (TG), TC, LDL-C and high-density lipoprotein cholesterol (HDL-C) levels were determined with commercial kits (Wako, Japan) by Hitachi 7020 full-automatic biochemical analyzer (Japan).

### Assay of plasma antioxidant capacity and lipid peroxidation

Superoxide dismutases (SOD) activity was estimated according to the method of Kono [14]. Catalase (CAT) activity was measured basing on the method of Goth [15]. Glutathione peroxidase (GPx) activity was determined by the method of Sazuka [16]. The glutathione (GSH) content was assayed by the method of Moron [17]. Thiobarbituric acid reactive substances (TBARS) level was assayed by the method of Buege [18]. The detection procedure of these enzymes activities has been described in detail in our preceding report [19].

### Assay of plasma inflammatory markers

The plasma interleukin 6 (IL-6) and C-reactive protein (CRP) levels were measured by means of commercially available Rat CRP ELISA kit (Abcam, Cambridge, MA) and Rat IL-6 ELISA kit (Abcam, Cambridge, MA), respectively. All the procedures and conditions were consistent with the instructions of these kits.

### Statistical analyses

Values are presented as mean ± SEM (standard error of the mean). The data were analyzed by one-way ANOVA, followed by the Fisher PLSD post hoc test if the overall differences were significant (*p* <0.05). All statistical analyses were performed using SPSS 13.0 statistical software (SPSS Inc., Chicago, IL) and a difference was considered significant when *p* <0.05.

## Results

### Plasma lipids

**Figure 1:**
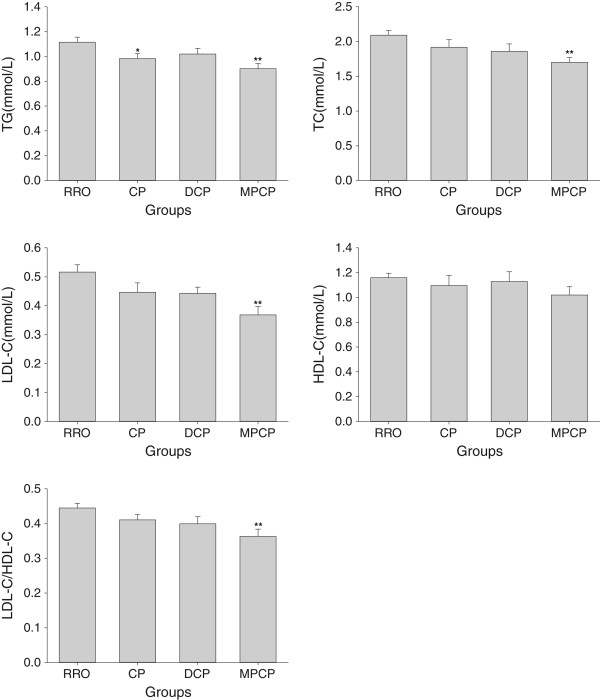
**Effects of optimized rapeseed oils on various plasma lipid parameters (TG, TC, LDL-C and HDL-C) and on the ratios of plasma LDL-C/HDL-C of rats fed a high-fat diet.** RRO: the refined rapeseed oil group; CP: cold pressing rapeseed oil group; DCP: dehulling-cold pressing rapeseed oil group; MPCP: microwave pretreatment-cold pressing rapeseed oil group. Bars represent the mean ± SEM from 10 animals in each group. *p <0.05 and **p <0.01 compared to the RRO group.

As can be seen in Figure 1, the plasma TG levels showed significant decreases in CP and MPCP groups than RRO group (*p* <0.05 and 0.01, respectively). Although compatible HDL-C levels in plasma were observed among all groups (*p* >0.05), animals in MPCP group exhibited marked decline in TC and LDL-C levels as well as the ratio of LDL-C/HDL-C in plasma compared with those in the RRO group (*p* <0.01).

### Plasma antioxidative capacity and lipid peroxidation

**Figure 2:**
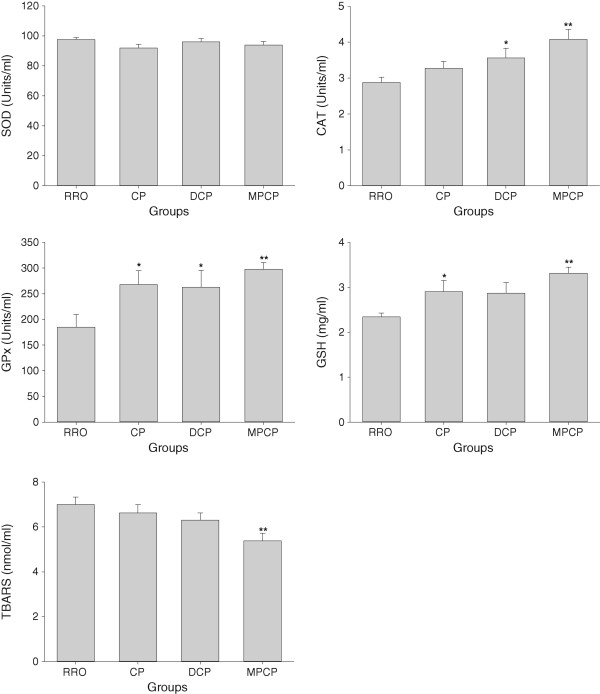
**Effects of optimized rapeseed oils on antioxidant enzymes (SOD, CAT and GPx) activities, GSH and TBARS contents in plasma of rats fed a high-fat diet.** RRO: the refined rapeseed oil group; CP: cold pressing rapeseed oil group; DCP: dehulling-cold pressing rapeseed oil group; MPCP: microwave pretreatment-cold pressing rapeseed oil group. Bars represent the mean ± SEM from 10 animals in each group. *p <0.05 and **p <0.01 compared to the RRO group.

As shown in Figure 2, there were no significant differences in plasma SOD activities among various rapeseed oil groups (*p* >0.05). Animals in CP, DCP and MPCP groups displayed significantly higher GPx activities (*p* <0.05, 0.05 and 0.01, respectively) and rats in DCP and MPCP groups had marked enhancement of CAT activities (*p* <0.05 and 0.01, respectively) when compared with their counterparts in RRO group. Besides, plasma GSH levels were also found to be elevated in CP and MPCP groups as compared to that in RRO group (*p* <0.05 and 0.01, respectively). When plasma TBARS were evaluated as the marker of lipid peroxidation, animals in MPCP group revealed markedly lower TBARS levels than that in RRO group (*p* <0.01).

### Plasma inflammatory

**Figure 3:**
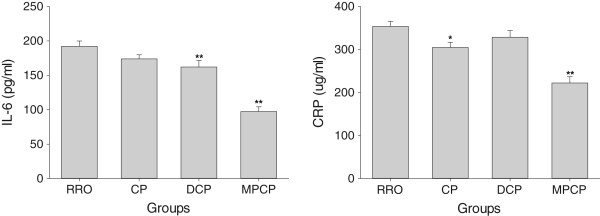
**Effects of optimized rapeseed oils on IL-6 and CRP levels in plasma of rats fed a high-fat diet.** RRO: the refined rapeseed oil group; CP: cold pressing rapeseed oil group; DCP: dehulling-cold pressing rapeseed oil group; MPCP: microwave pretreatment-cold pressing rapeseed oil group. Bars represent the mean ± SEM from 10 animals in each group. *p <0.05 and **p <0.01 compared to the RRO group.

When the plasma IL-6 and CRP levels were examined as the systemic markers for inflammation, both of them were affected by the micronutrient contents of experimental oils which have been shown in Figure 3. The plasma levels of IL-6 in DCP and MPCP groups (*p* <0.01) as well as CRP in CP and MPCP groups (*p* <0.05 and 0.01, respectively) were significantly lower than those in RRO group.

## Discussion

Although considerable progress has been made in the treatment of CVD with drug therapy over the past decades, this disease still remains the dominant epidemic in the world. It is widely understood that high fat diets, especially saturated fat, are implicated in the onset and development of CVD. High fat intake leads to plasma lipid abnormalities [20], including hypertriglyceridemia and hypercholesterolemia, oxidant stress [20] as well as low-grade system inflammation [21], which are major risk factors for CVD development [1–3].

After most of the erucic acid is removed by genetic engineering, the production and consumption of rapeseed oil leaped into prominence. Rapeseed oil ingestion has shown beneficial effects for CVD prevention. For example, replacing dairy fat with rapeseed oil can cause rapid and clinically relevant reductions in serum TG, TC, LDL-C and the ratio of LDL-C/HDL-C for hyperlipidaemic individuals [22]. The lipid-lowering efficacy of refined rapeseed oil is related to the sufficient amounts of alpha-linolenic acid (ALA) [23, 24]. ALA has been reported to suppress the expression and activities of many hepatic fatty acid syntheses such as fatty acid synthase (FAS), malic enzyme and glucose 6-phosphate dehydrogenase [25, 26]. On the other hand, ALA also sharply upregulates hepatic peroxisomal and mitochondrial fatty acid oxidation rate by increasing the expression and activities of a series of fatty acid oxidation enzymes [26, 27]. In addition, ALA has been shown to enhance hepatic LDL-receptor expression and cholesterol catabolism/output [28].

Processing technologies decisively and directly have an influence on the quantity of micronutrients in oils. In this study, all optimized rapeseed oils obtained with new processing technologies contain more micronutrients and it is noteworthy that MPCP made the greatest micronutrient retention in oil. These bioactive compounds act synergistically with each other and exert more potent biological effects in combination than as single nutrients. As predicted, the optimized rapeseed oils provided additional hypolipidaemic effects than refined rapeseed oil and these endogenous micronutrients should responsible for the apparent boon. Phenols have been shown to reduce plasma TG, TC and LDL-C by altering hepatic triglyceride assembly and secretion, cholesterol absorption and the processing of lipoproteins in plasma [11]. Phospholipids, another dramatically increased cardiovascular protective micronutrients in optimized rapeseed oils, have been consistently demonstrated to have the ability to reduce plasma triglyceride and cholesterol [29]. Phytosterols have a similar chemical structure with cholesterol but themselves are absorbed only in trace amounts [30], thus they inhibit cholesterol absorption including recirculating endogenous biliary cholesterol which is a key step in cholesterol elimination [30]. In addition, the hypolipidaemic effects of the phytosterols were also associated with the down-regulation of hepatic acyl CoA:cholesterol acytransferase activity [31] and the increasing LDL receptor expression [32].

The imbalance between the cellular free radical formation and the antioxidant defense leads to oxidative stress. The relative excessive production of free radicals can attack and denature many different cellular components, including lipids, proteins and DNA, which initiates the processes of atherogenesis through cell dysfunction [33]. In fact, oxidative stress is the unifying mechanism for many CVD risk factors [34]. For example, native LDL becomes oxidized in response to free radicals leading to the formation of oxidized LDL [12, 34] which plays an important role in the genesis and progression of atherosclerosis [12]. However, the deleterious effects of oxidative stress can be prevented by enzymatic and non-enzymatic antioxidant defense mechanism. In mammals, the most important antioxidant enzymes include SOD which converts superoxide to hydrogen peroxide, GPx and CAT which are responsible for converting hydrogen peroxide to water [35]. GSH is a very important non-enzymatic antioxidant, which can react directly with free radical or act an electron donor in the reduction of peroxides catalyzed by GPx [36]. The main phenolic compounds in rapeseed oil are sinapic acid and its derivatives [37]. Sinapic acid can efficiently scavenge free radicals through an electron donation mechanism [38]. Canolol (4-Vinylsyringol) is one of derivatives of sinapic acid and the mainly phenols in optimized rapeseed oils, which is more efficient as an radical scavenger than many other antioxidants, including α-tocopherol, vitamin C, β-carotene, rutin, and quercetin [39]. Tocopherol is also well known to acts as a powerful antioxidant by breaking chain reactions propagated by free radicals. Besides, the increased micronutrient contents in optimized rapeseed oils were accompanied with the marked increase of the plasma antioxidant enzymes CAT and GPx activities as well as GSH contents in the present study. All of these indicated that the micronutrients in optimized rapeseed oils had abilities to enhance the antioxidant defense system. As results, MPCP oil which possesses the highest micronutrients contents had appreciable ability to reduce plasma lipid peroxidation level. Similar positive effects were also observed by elevating micronutrients levels in oils with different manners [19, 40–42].

Recent advances in both the basic and clinical science have recognized the critical role of inflammation in all stages of atherosclerosis [43–45]. Various proinflammatory risk factors (oxidized LDL, infectious agents, etc.) can trigger the production of proinflammatory cytokines which contribute to development and progression of atherosclerosis. IL-6 and CRP are two of the most important proinflammatory cytokines, and both of which have been served as inflammatory markers for evaluation of atherosclerotic risk [12, 46–48]. The enhancement of micronutrients in optimized rapeseed oils in the present study tended to reduce the levels of plasma IL-6 and CRP, and further, significant decreases of both inflammation markers were observed with the consumption of MPCP oil. Both sinapic acid and canolol have been reported to suppresses the expression of many proinflammatory mediators including inducible nitric oxide synthase, cyclooxygenase-2, tumor necrosis factor-α, and interleukin-1β via NF-κB inactivation and thus exert their anti-inflammatory effects [49, 50]. Tocopherol also exerts anti-inflammatory properties by reducing many biomarkers of inflammation in atherosclerosis [12]. Besides, since free radicals mediate many signaling pathways which underlie vascular inflammation in atherogenesis [51], all these antioxidants exert actions as inflammation preventive agents via antioxidation.

In conclusion, the optimized rapeseed oils produced by some new processing technologies have the abilities to improve plasma oxidative stress, lipid profile and inflammation in high fat diet fed rats and these positive effects were more pronounced for MPCC oil due to the most abundant inherent micronutrients in this oil. These results suggested that the optimized rapeseed oils rich in endogenous micronutrients might contribute to prevent atherogenesis and make them very promising functional food in cardiovascular health promotion.
